# Employees and the National Insurance Act

**Published:** 1912-06-15

**Authors:** 


### NATIONAL INSURANCE ACT DEPARTMENT.

### EMPLOYEES AND THE NATIONAL INSURANCE ACT.

### XV.—The Domestic Staffs in Hospitals—(*continued*).

It is now clearly recognised that domestic servants have to be insured under the National Insurance Act, and mistresses and servants alike are asking the question, "How does it affect me?" The matter is no longer, so to speak, in the air—it is being reduced to a personal question. Some societies and many individuals are calling for a postponement of the operation of the Act. Very wisely, we think, the Government is resisting this demand. Further delay would only mean a further period of grace within which no substantial progress would be made, and, without doubt, the same rush of inquiries would set in in the last few days before any other date that might be substituted for July 15. The call for personal information is urgent and insistent, and we are endeavouring to take our part in answering it.

How the Act Affects the Employer.

The Act throws upon the employer, whether of one servant only or of many, the duty of seeing that the contributions are paid. This sounds very alarming to a mistress unused to Acts of Parliament, and we can well imagine that when confronted with such a statement she will have many questions to ask. To illustrate the matter in a personal way we take a number of hypothetical questions and furnish the answers. The mistress says, "What are the contributions to which you refer?" In ordinary cases they are 6d. a week for each female domestic servant, no matter what wage is paid.

"Have I to pay all this myself?" Yes, in the first instance, but you are given power under the Act to deduct a proportion of it from the wages of your servant.

"How much may I deduct from the wage?" If you provide board and lodging you may make a deduction equivalent to 3d. a week from the wage of each servant. You may also make the same deduction of 3d. a week from the wages of all servants under twenty-one years of age, even though board and lodging are not provided. If, however, you have a domestic servant who is over twenty-one years of age for whom you do not provide board and lodging, and whose wage does not exceed 1s. 6d. a day, you will not be able to make any deduction whatever from the wage. A small concession will be made to you in this case, as the contribution will be reduced to 5d. instead of 6d., the State paying the balance of 1d. In similar circumstances, when the wage is greater than 1s. 6d. a day, but does not exceed 2s. a day, the total contribution is again 5d., but you will be entitled to obtain 1d. a week by deduction from the servant's wage. When the wages are above 2s. a day the normal deduction of 3d. a week is allowed.

"Must I trouble about all this now—cannot you call again in three months' time when I have been able to think it over?" The Act comes into operation on July 15 next, and if you do not pay the contributions from that date onwards you will be liable to a penalty on conviction of £10 for each offence, and to pay the contributions that should have been paid. You may also have to make good any benefits the servant has lost through your negligence. Another ground for taking action as soon as the Act comes into operation is that unless the deductions just referred to are made from the particular payment of wages to which the contributions refer you are disallowed by the Act from making the deductions or requiring payment from the servant at any subsequent time.

"Tell me then, how are the payments to be made?" By means of stamps—not ordinary postage stamps, but special Health Insurance stamps.

"Where may these special stamps be obtained?" At any Post Office within the United Kingdom.

"What is to be done with them when they have been purchased?" The purchase of the stamps is not the end of the employer's duty; you are required to affix them to your servant's card, one for each week during any part of which she is employed.

"But what is to happen if my servant does not possess a card?" In such circumstances you must insist on a card being obtained by your servant, and if she resolutely refuses to act in the matter you will have to obtain a special emergency card from a Post Office to take the place of the usual member's card.

"Very well, I now understand this National Insurance Act is law. Now perhaps you will tell my servant what she has to do."

How the Act Affects the Servant.

The servant is told that she must obtain a card to which stamps will have to be affixed, and the most natural question that immediately comes to her lips is, "Where am I to get it from?" The first card may be obtained either from a Post Office or from an approved society.

"You say the *first* card—shall I have many cards to keep?" Each card will serve for a period not exceeding thirteen weeks, and thirteen spaces are provided, already dated, for the stamps representing those weeks' contributions. When the card is full you will not keep it, but you will have to send it to your society or to the Post Office, and a fresh card will then be issued for the next succeeding quarter.

"What do you mean by an approved society?" The Act is designed to furnish benefits in certain circumstances on the insurance plan, that is to say, advantage will be taken of the fact that while all contribute some will not have reason to claim the benefits, perhaps, for many years, and in consequence benefits very much larger than the amount of contributions paid by the individual member will be available for those who are entitled to them. All persons are liable to sickness and all stand equally to gain by such an arrangement. Further than this, as the State is making a special grant, in the case of women, of one quarter of the sum expended in benefits and in their administration, it is provided that only such societies as obtain the approval of the Insurance Commissioners shall be allowed to undertake the business.

"Why then do you mention the Post Office?" The approved societies are not bound to accept as members all servants who apply for membership. There are a number of servants who will be unable to obtain admission as members of an approved society on the ground of ill-health or for some other reason, and they will then be forced to become deposit contributors through the Post Office. In such case the servant will merely draw on the contributions paid on her behalf.

"Do you then advise me to join an approved society?" Most certainly. It is only by so doing that you will be able to obtain full benefits under the Act. Not only so, but your society will relieve you of all anxiety as to obtaining cards.

"How shall I choose a society?" Many societies are being formed, and the benefits receivable and contributions payable at the outset are the same in all of them. The time will come, however, when the well-managed society will be able to give better benefits than one that has been badly managed. In this matter a large society with an existing organisation is in a position to work more satisfactorily.

"Must I always belong to the same society? What is to happen if I go to a situation in another district?" You will be permitted to transfer from one society to another, and in certain circumstances you may be required to discontinue your membership. One special advantage of joining a society whose operations extend throughout the United Kingdom is that you will be able to continue your membership even though your work necessitates removal to another district. "What benefits shall I get?" In the first place you will be entitled to free medical attendance and treatment. Secondly, if you are so unfortunate as to be attacked by consumption you will be entitled to treatment in a sanatorium. Thirdly, when you are ill a payment will be made of 7s. 6d. a week if you are over twenty-one, or a smaller sum if under that age and you have no dependants. This benefit commences on the fourth day of sickness, and may be paid for a period of twenty-six weeks. If you had been over fifty years of age at the present time the benefit would have been at a reduced rate. Fourthly, if your sickness lasts longer than twenty-six weeks you will be entitled to a continuation of the payment at the rate of 5s. a week so long as the disablement continues. These last two benefits cease at the age of seventy. And fifthly, you will be entitled to a payment of 30s. as a maternity benefit.

"Do these benefits commence at once?" No, there are certain waiting periods—six months, for instance, in the case of sickness benefit. "Cannot I obtain something else instead?" Yes, it will be possible for a substitution to be made of one or more of the additional benefits specified by the Act. The most suitable form of benefit will probably be a superannuation allowance.

"Will you tell me the best way of settling the whole matter?" The first thing to do is to find a representative of an approved society, and to sign the form of proposal he will fill in according to your directions. He will ask you your age; and let me warn you here to be very careful to give this correctly, as there are severe penalties if it is incorrectly stated. This application for membership should be completed at once. You will not be required by so doing to pay anything before July 15, neither will your mistress.

---

### Answers to Correspondents.

### VALUATION OF SOCIETIES UNDER THE ACT.

"M. H." writes: "I understand that Scottish members of the Nurses' Insurance Society will, when societies come to be valued under the Act, be looked upon as a society separate from the English section, and that if the members in Scotland are under 5,000, certain disadvantages will thereby ensue. Can you tell me if this is so?"

*** The Nurses' Insurance Society will be a centralised society, but for the purpose of valuation the members resident in England, Scotland, Ireland, and Wales respectively will have to be treated as if they formed separate societies. If then the Scottish members should be less than 5,000 they will have to be associated, for the purpose of valuation, and the resulting disposal of surplus or deficiency in the stated proportions with other societies either of their own choosing or as may be arranged by the Insurance Commissioners, so that the total number of members in such association is at least 5,000. This is not necessarily a disadvantage; in certain circumstances it may even be an advantage, as, for instance, if it should happen that the Scottish nurses experienced unfavourable sickness rates resulting in a deficiency, whilst the other associated societies had a surplus. The number selected, namely, 5,000, while it may be large so far as Scottish nurses are concerned, is none too big to allow of the proper application of the principle of averages.

---

### THE FATE OF SMALL BENEFIT SOCIETIES.

"Clericus."—There are a great many benefit societies in the same position as the small ones in your village to which you refer—namely, too small to be registered—and it seems a great pity that they should all be disbanded. We are happy to inform you that we have made arrangements with an actuary to give advice in such cases and in respect to other similar difficulties in connection with the Insurance Act. If you will write fully, stating the circumstances, and address the letter to The Hospital Bureau of Information, 28 Southampton Street, Strand, London, W.C., you will receive a full reply to all queries and expert advice as to the best course to pursue under the circumstances.

---

NOTE:—Questions and Correspondence on all doubtful points are invited, and should be addressed to the Insurance Editor, The Hospital, 28 Southampton Street, Strand, W.C.

